# Investigation of the relationship between expression patterns of plasma microRNAs and pregnancy complications

**DOI:** 10.1590/1806-9282.20260435

**Published:** 2026-09-28

**Authors:** Nevin Karakus, Elif Donmezoglu Kutukcu, Sumeyye Yildirim

**Affiliations:** 1 Tokat Gaziosmanpasa University, Faculty of Medicine, Department of Medical Biology – Tokat, Turkey.

**Keywords:** Pregnancy complications, MicroRNAs, Real-time polymerase chain reaction

## Abstract

**OBJECTIVE:**

The aim of the study was to determine the association between plasma expression levels of microRNAs (miRNAs; MIR126-3p; and MIR375-3p) and pregnancy complications.

**METHODS:**

This prospective study included 39 pregnant women who admitted to Obstetrics and Gynecology Polyclinic of Tokat Gaziosmanpasa University, Faculty of Medicine. 28 of these pregnant women were healthy, while 11 had pregnancy complications (five hypertensive disorders of pregnancy and six gestational diabetes). Hypertensive disorders include preeclampsia, gestational hypertension, and chronic hypertension. The study included pregnant women older than 18 years of age with singleton pregnancies and no history of cancer. Quantitative real time-polymerase chain reaction was used to determine the expression patterns of MIR126-3p and MIR375-3p in maternal plasma. Fold change (2^-ΔΔCt^) analysis was used to examine the microRNA expression patterns.

**RESULTS:**

MIR375-3p expression levels demonstrated a statistically significant difference between two groups. It was significantly lower in women with pregnancy complications than healthy pregnant women.

**CONCLUSION:**

We found that MIR375-3p might be a useful indicator for non-invasive diagnosis and play a crucial part in predicting pregnancy complications. Given the limited patient cohort, more research using bigger samples is required to validate our findings.

## INTRODUCTION

The most important problems facing contemporary obstetrics are pregnancy complications, which impact at least 15% of pregnancies globally and include preeclampsia, gestational diabetes, and premature delivery^1^. They are linked to long-term negative outcomes for both the mother and the child, and they have a significant influence on maternal and fetal death and morbidity. Nonetheless, it is generally acknowledged that early detection of women at risk may enhance successful therapies, therefore lowering the prevalence of these potentially fatal disorders and the expense of newborn care.

MicroRNAs (miRNAs) are small non-coding RNAs that attach to mRNA, prevent translation, or encourage degradation in order to control gene expression. Since their discovery in 1993, miRNAs have been linked to essential cellular processes, including tissue development, proliferation, differentiation, apoptosis, and maintenance of homeostasis^2^. To prevent destruction, tissue miRNAs are released into the bloodstream, coupled to certain proteins or high-density lipoproteins, or encased in exosomes, microvesicles, or apoptotic bodies^2^. We refer to them as circulating miRNAs. These miRNAs play roles in vascular health, coagulation control, and immune tolerance during pregnancy.

The pregnancy complications have been linked to altered expression levels of circulating miRNAs that could be helpful in diagnosis and prognosis of these diseases^3,4^. Certain miRNAs carried by microvesicles have the ability to alter endothelial cell activity, which can affect vascular health and placental function^5–10^. Also, when compared to the normal placenta, the preeclampsia placenta exhibits abnormal expression of many miRNAs: some upregulated (MIR210 and MIR141) and some downregulated (MIR223 and MIR376c)^11–14^.

MIR126, a highly conserved miRNA, seems to be one of the most crucial miRNAs for healthy development. During pregnancy, MIR126, a well-known regulator of angiogenesis and endothelial function, is essential for preserving vascular integrity by limiting leukocyte adherence to the endothelium^15^. There have been reports of both increased and decreased MIR126 expression in placentas and serums of preeclampsia patients^15–18^. There are also studies that found increased expression of MIR375-3p in the plasma of preeclampsia patients^19,20^.

Because miRNAs in maternal blood are stable, they are perfect for non-invasive diagnostic testing in cases of pregnancy complications. Clinicians can learn more about the placental health of pregnant women and perhaps enable early intervention by examining their levels and activity^21^. The aim of the current study was to examine the association between the plasma expression levels of miRNAs (MIR126-3p and MIR375-3p) and pregnancy complications.

## METHODS

### Study samples

The ethical suitability of this study was confirmed by the Non-Intervention Scientific Research Ethics Committee of Tokat Gaziosmanpasa University Faculty of Medicine in April 8, 2025 (Registration No: 25-MOBAEK-116) and all the participants signed informed consent form for this study. This study included 39 pregnant women who visited the Obstetrics and Gynecology Polyclinic of Tokat Gaziosmanpasa University Faculty of Medicine. 28 of these pregnant women were healthy, while 11 had pregnancy complications (five hypertensive disorders of pregnancy and six gestational diabetes). Hypertensive disorders include preeclampsia, gestational hypertension, and chronic hypertension. After 20 weeks of pregnancy, new-onset hypertension (diastolic blood pressure [DBP] ≥90 mm Hg/systolic blood pressure [SBP] ≥140 mm Hg) is considered gestational hypertension. Women were presumed to have chronic hypertension if they had hypertension prior to 20 weeks of pregnancy. New-onset hypertension (DBP ≥90 mm Hg/SBP ≥140 mm Hg) with proteinuria or signs of end-organ damage after 20 weeks of pregnancy was considered preeclampsia. Gestational diabetes was diagnosed using a single-step approach. For this, a 75-g oral glucose tolerance test was performed in the morning after at least 8 h of fasting. The presence of at least one of the following: plasma glucose (fasting) values: ≥92 mg/dL, 1-h ≥180 mg/dL, 2-h ≥153 mg/dL was considered sufficient for a gestational diabetes diagnosis. The inclusion criteria for all the pregnant women was being older than 18 years of age with singleton pregnancies and having no history of cancer. Other inclusion criteria included having a hypertensive disorder for the patient group and not having any pregnancy complications for the control group. Exclusion criteria for all pregnant women include having intrauterine infection or any other surgical, medical or other obstetric complaint, a history of cancer, multiple pregnancy, and being under 18 years of age. For this study, a total of 38 samples were detected by using the G*Power 3.1.9.7 program with 78% power, 5% margin of error, and an effect size of 0.8.

### Sample preparation and the use of real-time polymerase chain reaction analysis

Five milliliters of maternal blood samples were placed in ethylenediaminetetraacetic acid tubes, which were centrifuged for 10 min at 3,000 rpm. Following centrifugation, the plasma was transposed to microcentrifuge tubes and kept at −80°C until it was required. The miRNAs in the plasma samples were extracted by following the manufacturer's instructions for the SanPrep Column microRNA Mini-Preps Kit (Code: SK8811, Biobasic, Canada). The complementary DNA (cDNA) conversion for both miRNAs was carried out using the miR-cDNA Synthesis kit found in the single-miRNA quantitative polymerase chain reaction (qPCR) test kit (Cat No: Q07-01-10, Atlas Biotechnologies, Türkiye), which was present independently for both miRNAs. The expression patterns of MIR375-3p and MIR126-3p in plasma were next ascertained by a RT-PCR using the obtained cDNAs and the miR-qPCR MasterMix that came with the single-miRNA qPCR test kit (Cat. No.: Q07-01-10, Atlas Biotechnologies, Türkiye). For reverse transcription and RT-PCR, respectively, the miR-cDNA Synthesis kit and miR-qPCR MasterMix include the following essential ingredients: RNase inhibitor, reverse transcriptase, antibody-mediated hot-start Taq DNA polymerase, miRNA-specific stem-loop primer, forward and reverse primers, MgCl_2_, nucleoside triphosphates (dNTPs), ROX reference dye, SBYR green dye, stabilizer, and enhancer. An Applied Biosystems 7500 Fast RT-PCR System (Applied Biosystems, Foster City, CA, USA) was the device that was used to apply RT-PCR. The comparative threshold cycle (Ct) method was used to evaluate each miRNA's relative expression. The calculations directly used the Ct values of the target genes and the reference gene (RNU6-1), which was used for normalization. Fold change (2^-ΔΔCt^) analysis was applied to analyze the miRNA expression patterns.

### Statistical analysis

The statistical analysis was carried out using IBM Statistical Package for the Social Sciences Statistical Package version 27.0. Characteristics of the samples were stated as either the mean ± standard deviation (SD) or the number, depending on availability. To assess the statistical value of circulating miRNAs, a one-way analysis of variance (ANOVA) test was employed. Different pregnancy complications and healthy pregnancies were compared pairwise using Tukey's test and one-way ANOVA. Using the boxplot method, the graphs were created. Statistical significance was defined as a two-tailed p-value of less than 0.05.

## RESULTS

This study was conducted with a total of 39 pregnant women, including 28 healthy pregnant women and 11 pregnant women with pregnancy complications. Of the 11 pregnant women with pregnancy complications, 6 had gestational diabetes, and 5 had hypertensive disorders (Table 1). The age and trimester distributions of pregnant women with pregnancy complications and healthy pregnant women were consistent (Table 1). Furthermore, no difference was seen between the two groups for parity, gravity, and abortion numbers. However, the SBP and DBP values differed between the two groups. SBP and DBP values were significantly elevated in pregnant women with pregnancy complications compared to healthy pregnant women as expected (Table 1).

**Table 1:** The demographical and clinical characteristics of pregnant women with pregnancy complications and healthy pregnant women.

Characteristics		Pregnant women with pregnancy complications (n=11)	Healthy pregnant women (n=28)	p-value
Age, years		30.96±1.347	32.70±4.165	0.056
Gestational age, weeks		29.18±5.250	31.64±7.041	0.302
Trimester, n (%)				
	2. Trimester (15–27 weeks)	4 (36.4)	8 (28.6)	0.411
	3. Trimester (28–33 weeks)	4 (36.4)	6 (21.4)	
	3. Trimester (34–40 weeks)	3 (27.3)	14 (50.0)	
Complication, n (%)				
Hypertensive disorders of pregnancy		5 (45.5)	-	-
Gestational diabetes		6 (54.5)	-	
SBP, mmHg		129.43±18.329	116.44±7.649	**0.008**
DBP, mmHg		80.429±10.014	72.72±7.525	**0.033**
Gravida, n		2.89±0.601	3.12±1.705	0.701
Parity, n		1.44±0.527	1.58±1.391	0.784
Abortion, n		0.44±0.726	0.54±0.905	0.780

Mean plus standard deviation values are presented for all the characteristics. BMI: body mass index; DBP: diastolic blood pressure; SBP: systolic blood pressure. The statistically significant values are represented in bold.

According to statistical analyses, while the expression levels of MIR126-3p did not differ between healthy pregnant women and pregnant women with pregnancy complications, the expression levels of MIR375-3p showed a statistically significant variation between these two groups (Table 2). The expression level of MIR375-3p was lower in pregnant women with pregnancy complications compared to healthy pregnant women. When pregnant women with pregnancy complications were separated into categories based on disease type, such as gestational diabetes and hypertensive disorders of pregnancy, and compared among themselves and with healthy pregnant women, no statistically significant association was observed between expression levels of both miRNAs and these categories (Table 3).

**Table 2:** Expression levels and p-values of MIR375-3p and MIR126-3p in pregnant women with pregnancy complications and healthy pregnant women.

miRNA	Group	n	Expression level (2^-ΔΔCt^)	p-value
MIR375-3p	Pregnant women with pregnancy complications	11	0.52±0.210	**0.015**
	Healthy pregnant women	28	1.56±1.321	
MIR126-3p	Pregnant women with pregnancy complications	11	2.89±1.250	0.403
	Healthy pregnant women	28	2.14±2.829	

miRNA: microRNA. The statistically significant values are represented in bold.

**Table 3:** Expression levels and p-values of MIR375-3p and MIR126-3p in pregnancies with different complications and healthy pregnancy.

Group	n	Expression level (2^-ΔΔCt^)	p-value		
		MIR375-3p	Hypertensive disorders of pregnancy versus Pregnancy diabetes	Hypertensive disorders of pregnancy versus Healthy pregnancy	Pregnancy diabetes versus Healthy pregnancy
Hypertensive disorders of pregnancy	5	0.46±0.205	0.986	0.136	0.153
Gestational diabetes	6	0.58±0.218			
Healthy pregnancy	28	1.56±1.321			
		**MIR126-3p**	**Hypertensive disorders of pregnancy versus Pregnancy diabetes**	**Hypertensive disorders of pregnancy versus Healthy pregnancy**	**Pregnancy diabetes versus Healthy pregnancy**
Hypertensive disorders of pregnancy	5	3.45±1.525	0.781	0.537	0.965
Gestational diabetes	6	2.43±0.830			
Healthy pregnancy	28	2.14±2.829			

## DISCUSSION

In this study examining the relationship between pregnancy complications and miRNA expression, a statistically significant association was found between expression levels of MIR375-3p and pregnancy complications. However, when we divided our patient group into two categories based on disease type, such as gestational diabetes and hypertensive disorders of pregnancy, this association disappeared. The possible explanation for this is that the number of patients in both categories decreased as a result of this division.

Widespread vasculogenesis, pseudovasculogenesis, and angiogenesis are all carried out by the growing placenta to aid in the development of the fetus. These mechanisms are mediated by angiogenic factors and trophoblast invasion. Preeclampsia, one of the hypertensive disorders of pregnancy, is thought to be caused by early failure of these mechanisms during pregnancy^22^. Angiogenesis signaling and endothelial cell vascular integrity are primarily promoted by MIR126. There have been reports of both increased and downregulated MIR126 expression in placentas and serums of preeclampsia patients^15–18^. However, in our study, we found no association, neither downregulation nor upregulation, between pregnancy complications, including preeclampsia, and MIR126 expression.

In recent years, MIR375 has been widely reported in a range of immunological activities, indicating that it may be an important regulator of cell differentiation and an integral part of immune system regulation^23^. There were two reports that examined the association between preeclampsia and MIR375 expression^19,20^. Both studies found increased expression of MIR375-3p in the plasma of preeclampsia patients. These findings differ from ours. In our findings, MIR375 expression was decreased in pregnant women with pregnancy complications, but this association disappeared when we grouped the patients as having gestational diabetes and hypertensive disorder of pregnancy. This may be explained by variances in the populations under study and sample size as well as changes in pre-analytical and analytical procedures. Furthermore, because miRNAs are involved in epigenetic processes, additional variables including maternal age, lifestyle, illnesses, or drug misuse may have an impact on their expression levels. Thus, it is important to look into the effects of patient-to-patient variation in plasma miRNA levels.

The primary constraint of the current study was the limited sample size. In all, 39 samples were examined. Larger sample numbers are essential for identifying subtle but physiologically meaningful changes in miRNA research. Therefore, it's possible that this study's limited sample size prevented it from finding correlations found in earlier research. Furthermore, the restricted statistical power resulting from the small sample size may be the reason why there was no difference in the expression patterns of these miRNAs between the hypertensive diseases of pregnancy and gestational diabetes. Even evaluating miRNA expression levels by itself is insufficient to comprehend the intricacy of pregnancy complications.

In conclusion, we found a statistically significant association between expression levels of circulating MIR375-3p and pregnancy complications. These findings should be validated in larger, multi-center cohorts. Most significantly, validation is necessary in connection to clinical symptoms in order to evaluate the clinical importance of circulating miRNAs in pregnancy complications and to ascertain sensitivity, specificity, and predictive value. To enhance health care for all pregnant women in the future, it is likely that a panel of several biomarkers, including genomic, transcriptomic, proteomic, and biophysical ones, paired with maternal features, will be required due to the multifactorial nature of these illnesses.

## Data Availability

The datasets generated and/or analyzed during the current study are available from the corresponding author upon reasonable request.
